# Surgery

**Published:** 1876-11

**Authors:** 


### III. Surgery.

1. *On Ainhum.* Camuset. (*L'Union Méd*, August 26.)

A negro, forty years old, had a small furrow form on the inferior and internal surface of the little toe of the right foot, which subsequently became deeper, and completely surrounded the root of the toe, while the extremity enlarged. Finally strangulation occurred, and the toe becoming very sensitive and movable, was attached to the foot by a slender cord merely. This was divided by a stroke of the scissors, when a cure was effected.

When examined, upon admission, the toe was retracted, enlarged to three times its ordinary size, and rotated outward. A deep circular furrow, involving the entire thickness of the toe, completely surrounded the member at the digito-plantar root, leaving only the central pedicle, discovered by separating the two surfaces which were in contact. These surfaces were completely covered by integument, which was slightly retracted, smooth and normal, without perceptible cicatrix. Upon each of these surfaces was a slight elevation, giving issue to an extremely fetid and sero-purulent secretion. The pedicle was found to be constituted, not by the phalanx, but by a fibrous tissue, having on its surface a trace of the small ulcer mentioned above. When the section was made, the parts divided were seen to be uniformly whitish-yellow in color, especially at the center, and possessing an elasticity, due apparently to the connective and fatty tissue.

"Ainhum" was first described in 1867, by Silva Lima. It occurs exclusively among negro field laborers, and always upon the little toe. The case given above is in all respects typical.

The disease is chronic — lasts from one to ten years — etiology obscure. In Brazil men only are affected, but in Africa women are not exempt. The wearing of shoes seems not to offer immunity. The general health is unaffected by the disease. Moncerro, who treated the patient whose case is detailed, summarizes the points of difference between "ainhum" and the Norwegian "radezyge," "spedalskhed," elephantiasis, lepra, scleroderma, "bouba" (a disease analogous to the "yaws"), and symmetrical gangrene of the extremities.

He concludes that "ainhum" is a special disease, belonging to the degenerations described by Cullerier as "atrophies by rarefaction." In this the compact bony tissue becomes spongy and areolar, and is finally replaced by mere connective tissue and fat.

2. *Excision of Nerves, and their Reunion by Sutures.* Braun. (*Centralbl. f. Chir.*, 1876, 34.)

A young farmer received a punctured wound in his left arm. The bleeding, which had been quite profuse, ceased spontaneously; the small wound healed, but the fingers of the left hand remained paralyzed. Ten months after the injury, when the patient was admitted to the hospital at Heidelberg, several painful tumors of the size of cherry stones were discovered under the cutaneous cicatrix, which was located in the upper third of the arm, at the inner border of the biceps muscles. The left hand was cold, its integument thin, glistening, brown-red, and the finger nails were thickened, roughened, and much incurved, like claws. The interosseous muscles and the muscles of the thumb and little finger were atrophied. The result of the electric examination and the character of the palsy proved conclusively that the ulnar and median nerves had been completely cut through

Prof. Simon resolved on the attempt at reuniting the severed nerves. The operation was performed under antiseptic measures, and with the use of Esmarch's bandage. The central ends of the nerves, which were first exposed, formed club-like enlarged lumps. The distal ends of the nerves had retracted to such an extent that with great difficulty only they could be found. These were pared off; the swollen central ends were excised, and both ends were made to meet by means of a suture of finest silk, which passed through the entire thickness of the nerve. But in order to insure an accurate apposition of the cut surfaces, additional stitches of cat-gut were applied. The median nerve received two, the ulnar nerve three sutures. The tension was relieved by placing the arm upon a rectangular splint. For two days there was violent pain in the wound, which, after profuse suppuration, healed at the end of four weeks. The stitches were not removed till six weeks after the operation. The palsy then still existed, but the electric current applied above the cicatrix caused feeble contractions of the muscles of the hand. And eighteen months after this nerve-suture the patient showed this remarkable result:

The forearm is stronger, its skin thicker, warmer, and no more glistening; the finger nails are deformed no more. The function of the flexor and pronator muscles is completely restored, while the muscular actions of the thumb and little finger are still defective. The sensation has returned, though it is yet somewhat inferior to that of the right hand. The patient is perfectly able again to do his work.

Prof. von Langenbeck, at the last congress of German surgeons, reported a similar resection and reunion of nervous fibres which he performed on the sciatic nerve, with a very good result, two years after this nerve had been cut by a hatchet.

